# Regulation of mitochondrial oxidative stress by β-arrestins in cultured human cardiac fibroblasts

**DOI:** 10.1242/dmm.019968

**Published:** 2015-12-01

**Authors:** Jennifer L. Philip, Md. Abdur Razzaque, Mei Han, Jinju Li, Tiju Theccanat, Xianyao Xu, Shahab A. Akhter

**Affiliations:** 1 Department of Surgery, Division of Cardiothoracic Surgery, University of Wisconsin School of Medicine and Public Health, Madison, WI 53792, USA; 2 Section of Cardiac and Thoracic Surgery, University of Chicago Medical Center, Chicago, IL 60637, USA

**Keywords:** β-arrestin, Oxidative stress, NADPH oxidase, Heart failure, Cardiac fibroblast, Collagen, Myocardial fibrosis

## Abstract

Oxidative stress in cardiac fibroblasts (CFs) promotes transformation to myofibroblasts and collagen synthesis leading to myocardial fibrosis, a precursor to heart failure (HF). NADPH oxidase 4 (Nox4) is a major source of cardiac reactive oxygen species (ROS); however, mechanisms of Nox4 regulation are unclear. β-arrestins are scaffold proteins that signal in G-protein-dependent and -independent pathways; for example, in ERK activation. We hypothesize that β-arrestins regulate oxidative stress in a Nox4-dependent manner and increase fibrosis in HF. CFs were isolated from normal and failing adult human left ventricles. Mitochondrial ROS/superoxide production was quantitated using MitoSox. β-arrestin and Nox4 expressions were manipulated using adenoviral overexpression or short interfering RNA (siRNA)-mediated knockdown. Mitochondrial oxidative stress and Nox4 expression in CFs were significantly increased in HF. Nox4 knockdown resulted in inhibition of mitochondrial superoxide production and decreased basal and TGF-β-stimulated collagen and α-SMA expression. CF β-arrestin expression was upregulated fourfold in HF. β-arrestin knockdown in failing CFs decreased ROS and Nox4 expression by 50%. β-arrestin overexpression in normal CFs increased mitochondrial superoxide production twofold. These effects were prevented by inhibition of either Nox or ERK. Upregulation of Nox4 seemed to be a primary mechanism for increased ROS production in failing CFs, which stimulates collagen deposition. β-arrestin expression was upregulated in HF and plays an important and newly identified role in regulating mitochondrial superoxide production via Nox4. The mechanism for this effect seems to be ERK-mediated. Targeted inhibition of β-arrestins in CFs might decrease oxidative stress as well as pathological cardiac fibrosis.

## Abstract (precis)

**Summary:** β-arrestins regulate oxidative stress in a Nox4-dependent manner leading to increased extracellular-matrix protein synthesis by cardiac fibroblasts (CFs). Targeted inhibition of β-arrestins in CFs might decrease pathological fibrosis.

## INTRODUCTION

Cardiac fibroblasts (CFs) make up 60-70% of the total cell number in the human heart and play a crucial role in regulating normal myocardial function through maintenance of extracellular matrix (ECM) homeostasis (Weber, 2004). Additionally, CFs have a key role in heart failure (HF) because they mediate the process of maladaptive remodeling. During this process, normally quiescent CFs undergo phenotypic modulation to activated myofibroblasts (Petrov et al., 2002). Myofibroblasts express α-smooth muscle actin (α-SMA), indicating acquisition of a secretory and contractile phenotype, a transition that correlates with increased synthesis and deposition of ECM proteins, including collagen (Porter and Turner, 2009; Baudino et al., 2006). Transforming growth factor-β (TGF-β) is the most potent profibrotic stimulus for CFs, and myocardial TGF-β levels are elevated in HF. TGF-β stimulates both CF-to-myofibroblast transformation and marked increases in ECM deposition (Brown et al., 2005).

Oxidative stress is thought to play a crucial role in the development and progression of cardiac remodeling associated with HF (Kinugawa et al., 2000; Sia et al., 2002). Reactive oxygen species (ROS) modulate ECM remodeling by mediating CF function and stimulating collagen turnover (Murdoch et al., 2006). NADPH oxidases (Nox) are the major source of ROS in the heart (Octavia et al., 2012), and Nox2 and Nox4 are the primary isoforms expressed in the myocardium (Byrne et al., 2003). Nox4 is constitutively active, localized to the mitochondria, and a major source of superoxide generation in the heart and specifically in CFs (Murdoch et al., 2006). It has recently been demonstrated that Nox4 mediates TGF-β-stimulated myofibroblast transformation in normal human CFs (Cucoranu et al., 2005). Mechanisms by which Nox4-mediated ROS production is regulated remain unclear.

β-arrestins are scaffold proteins involved in G protein-coupled receptor (GPCR) desensitization and downregulation, and also link GPCR activation to downstream signaling pathways including MAPK/ERK, PI3K and AKT (Lefkowitz et al., 2006). β-arrestins are crucial in the impaired myocardial β-adrenergic receptor signaling that is a hallmark of chronic HF, because β-arrestins target these receptors for internalization and degradation (Kelly et al., 2008). The potential role of β-arrestins in regulating oxidative stress in fibroblasts has not been previously studied.

In this study, we investigate the role of Nox4-mediated mitochondrial oxidative stress in adult human CF biology in the setting of chronic HF. We hypothesize that β-arrestins might regulate Nox4-mediated mitochondrial ROS production and play a substantial role in the development of the failing CF phenotype.

**TRANSLATIONAL IMPACT**

**Clinical issue**

The incidence of heart failure (HF) continues to increase worldwide, and effective medical therapies are currently lacking. Adverse remodeling of the heart is associated with cardiac myocyte hypertrophy and dysfunction, as well as myocardial fibrosis. This process of ventricular fibrosis is mediated by cardiac fibroblasts (CFs), which make up almost 70% of the total cell number in the human heart. Fibrosis initially leads to stiffening of the ventricle and diastolic dysfunction, and this can ultimately progress to severe systolic HF. The mechanisms that underlie the transformation of quiescent CFs to activated myofibroblasts that synthesize and deposit excess extracellular matrix (ECM) are just beginning to be understood. Oxidative stress has been implicated as one stimulus for this process. The focus of this study is to explore how oxidative stress is regulated in adult human CFs and to target this pathway as a potentially novel therapeutic strategy.

**Results**

Mitochondrial NADPH oxidases, particularly the isoform Nox4, are a major source of reactive oxygen species (ROS) production in the heart. The authors compared levels of ROS (in the form of mitochondrial superoxide) in CFs isolated from normal and failing adult human left ventricle. They show that ROS levels, together with Nox4 expression, are significantly increased in CFs in the context of failing left ventricles. Nox4 knockdown resulted in significant inhibition of mitochondrial superoxide production and decreased basal and TGF-β-stimulated α-SMA and collagen expression. β-arrestin expression in CFs was shown to be upregulated fourfold in failing CFs. β-arrestin knockdown decreased ROS and Nox4 expression by 50% in failing CFs. Conversely, β-arrestin overexpression in normal CFs increased mitochondrial superoxide production twofold. These effects were inhibited by inhibition of Nox or ERK. Upregulation of Nox4 seems to be a primary mechanism for increased ROS production in failing CFs, and this, in turn, stimulates ECM synthesis. β-arrestin expression is also upregulated in failing CFs, and plays an important role in regulating mitochondrial superoxide production via Nox4 in a mechanism that seems to be ERK-mediated.

**Implications and future directions**

This study provides the first evidence that β-arrestins can regulate oxidative stress in adult human CFs via Nox4 in an ERK-dependent manner. Furthermore, the work demonstrates that both TGF-β and β-arrestin signaling through ERK promotes an increase in Nox4 expression and mitochondrial ROS generation. Targeted inhibition of β-arrestins in CFs might thereby decrease oxidative stress and the pathological fibrosis that ensues in cardiac tissue. Further investigation of specific mechanisms for the ERK-mediated increase in Nox4 expression is underway and *in vivo* fibroblast-specific inhibition of β-arrestins will be studied as a potential therapeutic strategy to prevent adverse ventricular remodeling. These findings could also have potential therapeutic implications for other organ systems that develop pathological fibrosis, including lung, liver and kidney tissues.

## RESULTS

### Mitochondrial superoxide production and Nox4 are upregulated in failing cardiac fibroblasts

**Fig. 1.**
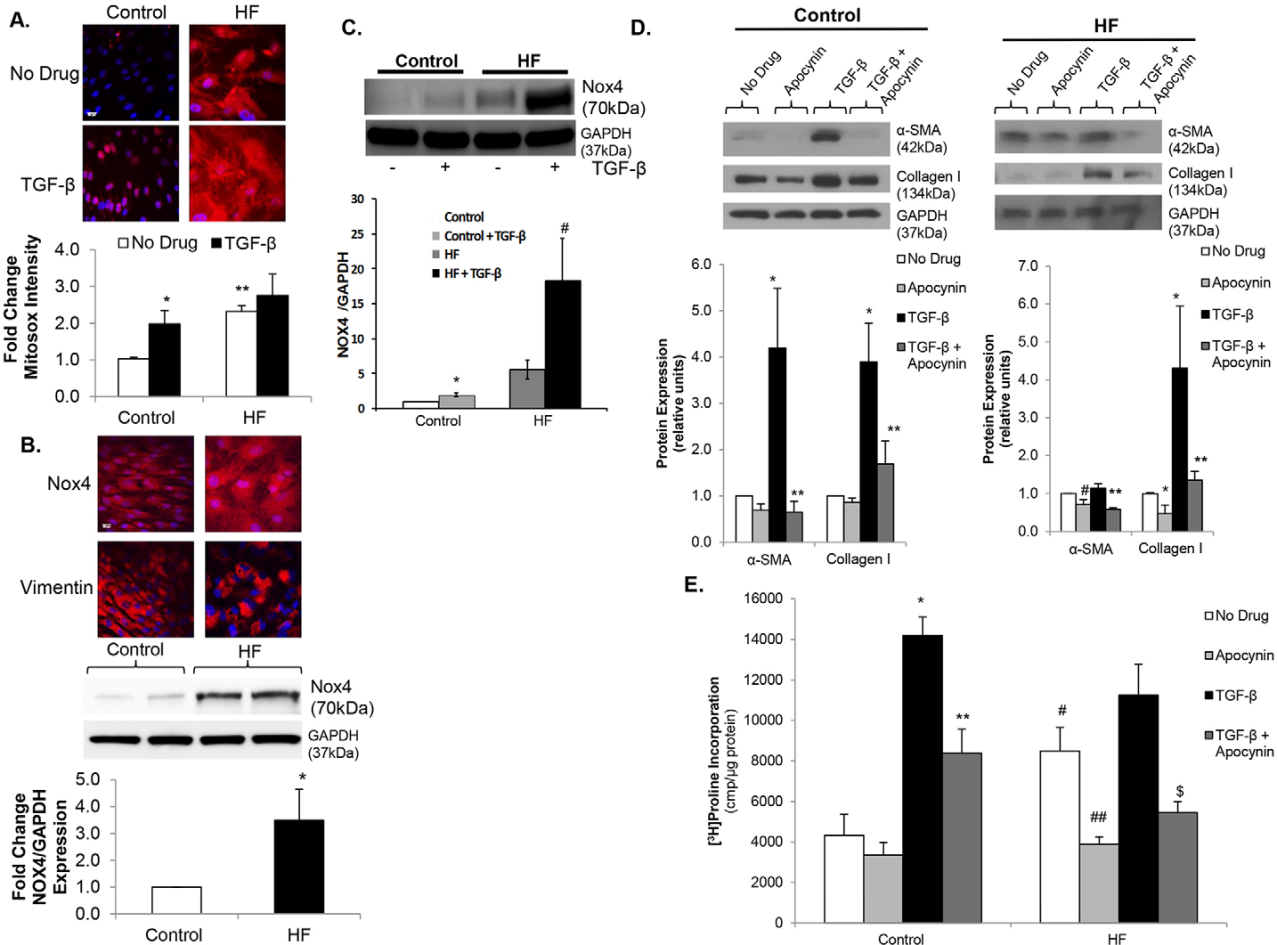
**Mitochondrial superoxide production and Nox4 are upregulated in failing cardiac fibroblasts.** (A) Confocal images (upper panel) of control and heart failure (HF) cardiac fibroblasts (CFs) stained with MitoSOX (red) under basal conditions (No Drug) vs TGF-β stimulation. Nuclei are stained blue with Hoechst 33342. Fluorescence quantitation shown below demonstrates an over twofold increase in mitochondrial oxidative stress in control CFs in response to TGF-β. *P<0.03 vs Control+No Drug; **P<0.005 vs Control+No Drug; *n*=3-4 in all groups. Scale bar: 20 μm. (B) Confocal images (upper panel) of Nox4 stained red with Alexa-Fluor-594 dye are show in in HF versus control CFs. Nuclei are stained blue with DAPI. Representative immunoblot (middle panel) showing Nox4 expression in HF versus control CFs. GAPDH was used as a loading control. Densitometric analysis (lower panel) demonstrates an over fourfold increase in Nox4 expression in HF. *P<0.05 vs Control; *n*=4 in all groups. This membrane was stripped, re-probed and quantitated in Fig. 3A. Scale bar: 20 μm. (C) Representative immunoblot (upper panel) showing Nox4 expression under basal conditions and following TGF-β stimulation in control and HF CFs. GAPDH was used as a loading control. Densitometric analysis (lower panel) demonstrates increased Nox4 expression in response to TGF-β in both control and HF. *P<0.05 vs No Drug; ^#^*P*<0.05 vs No Drug; *n*=5 in all groups. (D) Representative immunoblots (upper panels) showing the effects of apocynin on basal and TGF-β-stimulated α-SMA and collagen I expression in both control (left) and HF (right) CFs. GAPDH was used as a loading control. Densitometric analysis shown below. *P<0.02 vs No Drug, **P<0.03 vs TGF-β, ^#^*P*<0.05 vs No Drug; *n*=3-4 for all groups. (E) Collagen synthesis in HF versus control CFs under basal conditions, TGF-β stimulation, apocynin treatment, and pre-treatment with apocynin prior to stimulation with TGF-β. *P<0.001 vs Control+No Drug; **P<0.002 vs Control+TGF-β; ^#^*P*<0.003 vs Control+No Drug; ^##^*P*<0.01 vs HF+No Drug; ^$^*P*<0.002 vs HF+TGF-β; *n*=3 in all groups.

It is well established that oxidative stress is increased in the myocardium in the setting of HF. Markers of oxidative stress are increased in human HF, and these correlate with disease severity. We examined whether oxidative stress was specifically increased in human CFs isolated from failing left ventricles (LVs) compared to normal controls. CFs were stained with MitoSox to quantitate mitochondrial superoxide generation. There was a greater than twofold increase in mitochondrial superoxide levels in failing CFs versus control (Fig. 1A). We quantitated Nox4 protein expression in HF versus control CFs as a potential mechanism for the increased mitochondrial superoxide production. There was more than a threefold increase in Nox4 expression in failing CFs as demonstrated by immunostaining and immunoblotting (Fig. 1B). Because Nox4 is constitutively active, this increase in expression seems to be an important mechanism of increased mitochondrial superoxide levels in failing CFs. It is well established that TGF-β is an important profibrotic stimulus for CFs in both the healthy and failing myocardium (Weber, 2004; Petrov et al., 2002). We investigated whether TGF-β stimulation increases mitochondrial superoxide production. TGF-β stimulation increased MitoSox staining in normal CFs (Fig. 1A) to levels similar to those observed in failing CFs. In failing CFs, there was no additional increase in MitoSox fluorescence intensity following TFG-β stimulation (Fig. 1A). Additionally, TGF-β significantly increased Nox4 expression in both control and failing CFs (Fig. 1C). These data are consistent with previous findings showing a link between TGF-β and Nox4 expression in normal human CFs, and further demonstrate that mitochondrial superoxide production and Nox4 expression increase with activation of TFG-β signaling in human CFs.

### Increased oxidative stress contributes to adverse remodeling in CFs

The adverse remodeling phenotype observed in failing CFs is characterized by increased myofibroblast transformation and ECM production. To determine whether increased superoxide production contributes to this maladaptive process, we utilized the non-specific Nox inhibitor apocynin. Pre-treatment with apocynin led to significant inhibition of TGF-β-stimulated α-SMA expression, a marker of myofibroblast differentiation, in both control and failing CFs, as well as a significant reduction in basal α-SMA expression in failing CFs (Fig. 1D). Consistent with inhibition of myofibroblast differentiation, apocynin pre-treatment decreased basal and TGF-β-stimulated collagen expression as measured by western blot (Fig. 1D) and collagen synthesis as determined by [^3^H]proline incorporation (Fig. 1E). These data provide evidence that mitochondrial oxidative stress in fibroblasts is increased in HF and that increased oxidative stress contributes to CF-mediated adverse remodeling.

### Nox4 mediates TGF-β-driven myofibroblast transformation and mitochondrial superoxide production in human CFs

**Fig. 2.**
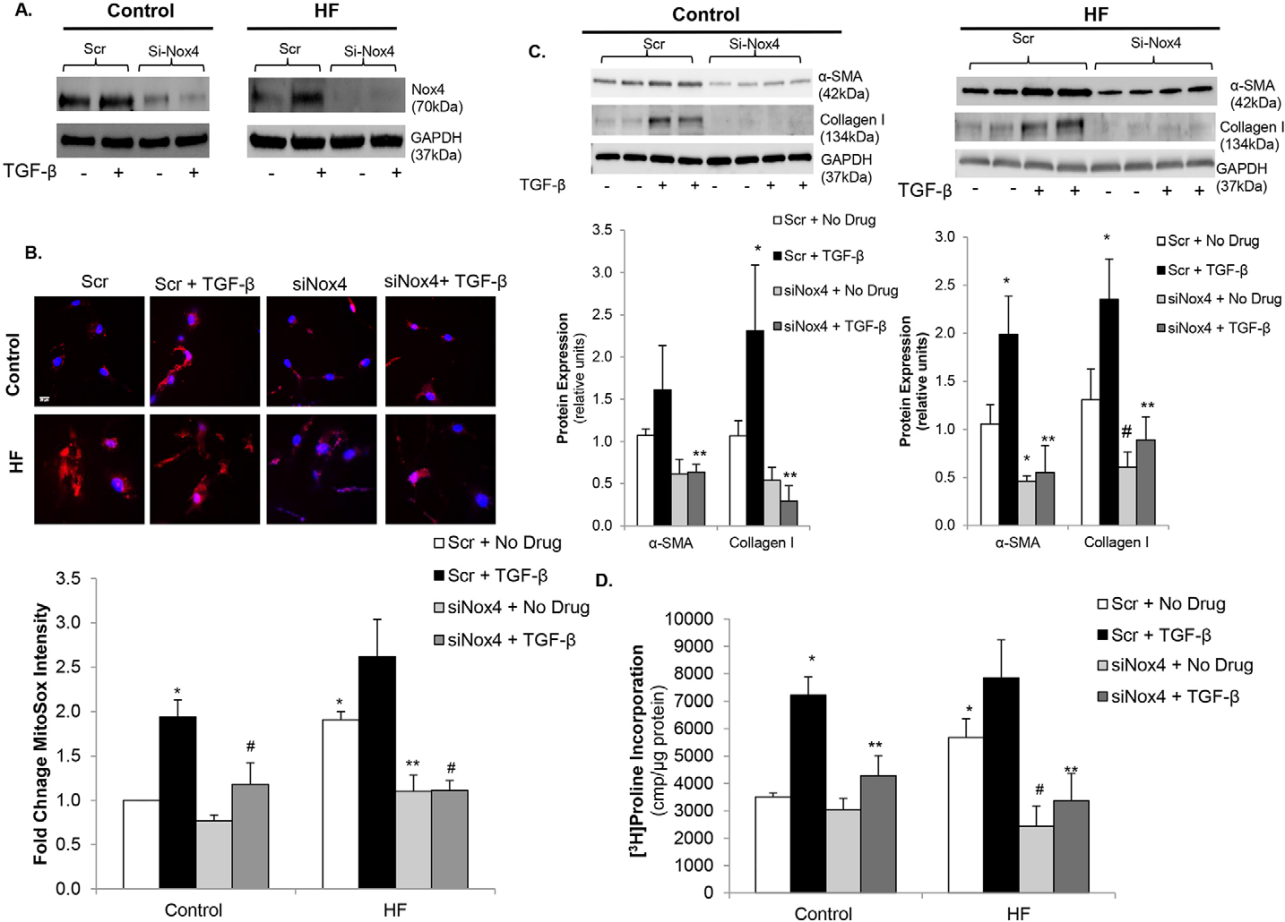
**Nox4 mediates myofibroblast transformation and collagen synthesis via increased oxidative stress.** (A) Representative immunoblots showing knockdown of Nox4 expression in control (left panel) and heart failure (HF) cardiac fibroblasts (CFs) (right panel) with *Nox4* siRNA (siNox4) vs scrambled control (Scr). GAPDH was used as a loading control. (B) Confocal images of CFs stained with MitoSOX (red) showing inhibition of mitochondrial oxidative stress with siNox4 in control and HF CFs. Nuclei are stained blue with Hoechst 33342. Fluorescence quantitation is shown below those images. *P<0.003 vs Control Scr+No Drug, **P<0.005 vs Scr+No Drug, ^#^*P*<0.02 vs Scr+TGF-β; *n*=3-4 in all groups. Scale bar: 20 μm. (C) Representative immunoblots (upper panels) showing the effects of Nox4 knockdown on basal and TGF-β-stimulated α-SMA and collagen I expression in control (left panel) and HF (right panel) CFs. GAPDH was used as a loading control. Densitometric analysis is shown below. *P<0.05 vs Scr+No Drug, **P<0.01 vs Scr+TGF-β, ^#^*P*=0.05 vs Scr+No Drug; *n*=3-4 in all groups. (D) Basal and TGF-β-stimulated collagen synthesis in control and HF CFs following transfection with siNox4 or Scr control. *P<0.05 vs Control Scr+No Drug, **P<0.005 vs Scr+TGF-β; ^#^*P*<0.01 vs HF Scr+No Drug; *n*=3-4 in all groups.

To more specifically investigate the role of Nox4 in TGF-β-stimulated mitochondrial superoxide production and CF-mediated adverse remodeling, we used a short interfering RNA (siRNA) approach to knockdown Nox4 expression. Inhibition of Nox4 protein expression was demonstrated by immunoblotting (Fig. 2A). In control CFs, siRNA knockdown of *Nox4* (siNox4) significantly inhibited TGF-β-stimulated mitochondrial superoxide production compared to scrambled control siRNA (Scr) (Fig. 2B). Nox4 knockdown in HF CFs returned superoxide production to control levels under basal conditions as well as following TGF-β stimulation (Fig. 2A). To determine whether Nox4 contributed to CF-mediated myocardial fibrosis, α-SMA expression and collagen production were examined after Nox4 knockdown. siNox4 led to significant inhibition of TGF-β-stimulated increases in α-SMA and collagen I protein expression in control CFs (Fig. 2C), providing evidence that TGF-β-driven myofibroblast differentiation might be Nox4-dependent. In failing CFs, both basal and TGF-β-stimulated α-SMA and collagen I expression were inhibited by siNox4 versus Scr (Fig. 2C), demonstrating that increased Nox4 expression might contribute to the maladaptive adverse remodeling phenotype in HF. Furthermore, Nox4 knockdown blunted TFG-β-stimulated collagen synthesis in both control and failing CFs (Fig. 2D). Interestingly, knockdown of Nox4 decreased collagen synthesis in the failing CFs to near control levels. These studies demonstrate that increased oxidative stress in the setting of HF and also following TGF-β stimulation seems to be mediated, to a large degree, by Nox4 and that knockdown of Nox4 reverses the activated profibrotic phenotype of failing CFs.

### Upregulation of β-arrestins in fibroblasts contributes to increased mitochondrial oxidative stress

**Fig. 3.**
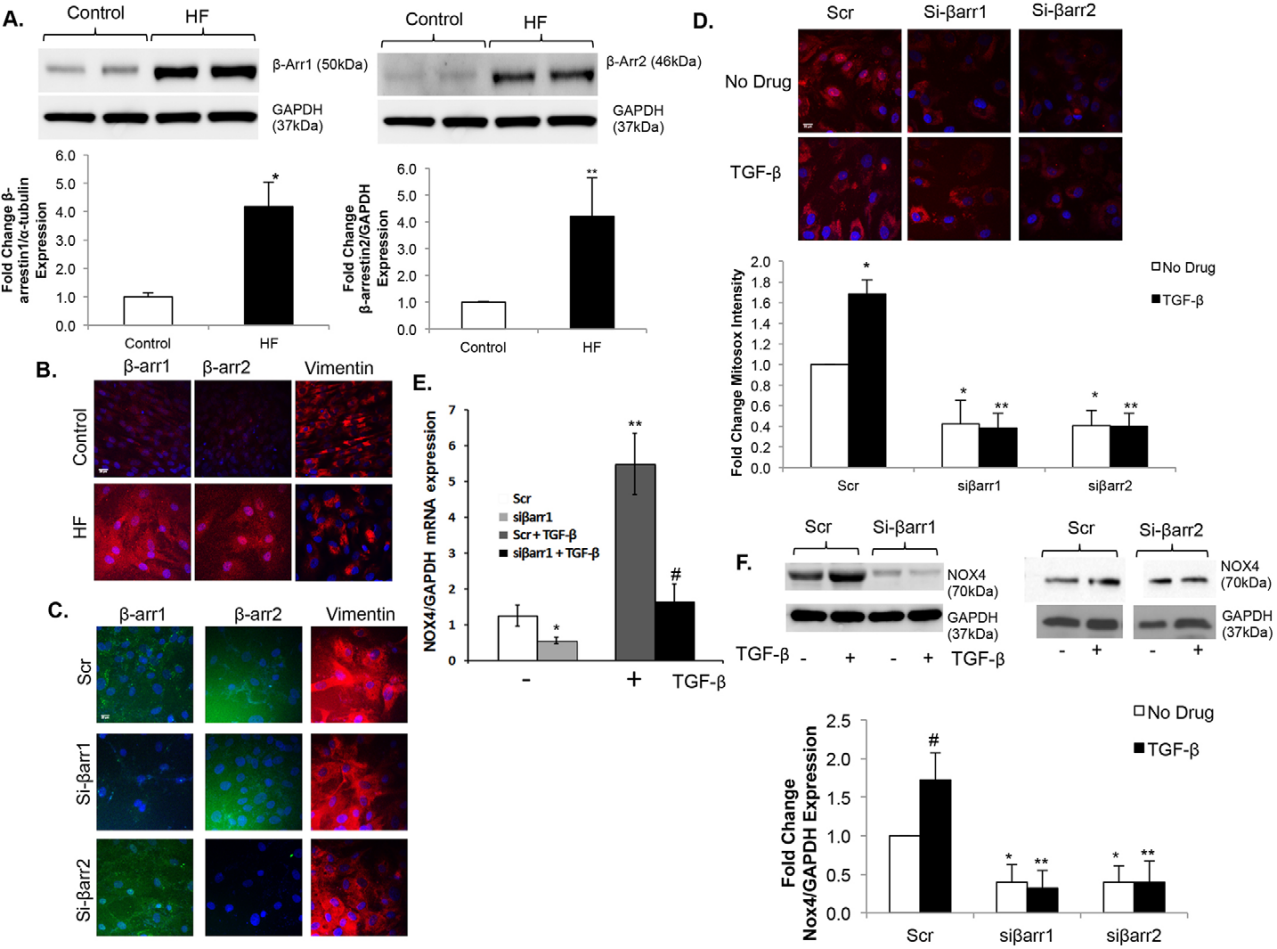
**β-arrestins regulate mitochondrial superoxide production and Nox4 expression in cardiac fibroblasts.** (A) The blot presented in Fig. 1B was stripped and re-probed (upper panels) for β-arrestin-1 (β-Arr1) (left) and β-arrestin-2 (β-Arr2) (right), showing expression in control vs heart failure (HF) cardiac fibroblasts (CFs) with the same GAPDH loading controls. Densitometric analysis (lower panels) demonstrates an over fourfold increase in β-arrestin-1 and β-arrestin-2 expression in HF. *P<0.01 vs Control; **P<0.04 vs Control; *n*=4 for β-arrestin-1 expression groups, *n*=5 for β-arrestin-2 expression groups. (B) Confocal images of β-arrestin-1, β-arrestin-2 and vimentin stained red with Alexa-Fluor-594 dye are shown in HF versus control CFs, demonstrating increased β-arrestin expression in HF. Nuclei are stained blue with DAPI. Scale bar: 20 μm. (C) Confocal images showing β-arrestin-1 and β-arrestin-2 stained green with FITC, and vimentin stained red with Alexa-Fluor-594 dye in HF CFs following siRNA knockdown of β-arrestin-1 (siβarr1), β-arrestin-2 (siβarr2) or scrambled control (Scr), demonstrating successful siRNA knockdown of β-arrestins. Nuclei are stained blue with DAPI. Scale bar: 20 μm. (D) Confocal images (upper panels) of failing CFs stained with MitoSOX (red) following siRNA knockdown of β-arrestin-1 (siβarr1), β-arrestin2 (siβarr2) or scrambled control (Scr). Nuclei are stained blue with Hoechst 33342. Fluorescence quantitation is shown below. *P<0.03 vs Scr+No Drug; **P<0.001 vs Scr+TGF-β; *n*=3 for all groups. Scale bar: 20 μm. (E) Quantitative PCR showing basal and TGF-β-stimulated *Nox4* mRNA expression following siβarr1 vs Scr control. Values are normalized to GAPDH. *P<0.03 vs siβarr1, **P<0.05 vs Scr+TGF-β, ^#^*P*<0.05 vs siβarr1+TGF-β; *n*=3 for all groups. (F) Representative immunoblot (upper panels) showing basal and TGF-β-stimulated Nox4 expression following siβarr1 or siβarr2 vs Scr control. GAPDH was used as a loading control. Densitometric analysis is shown below. *P<0.05 vs Scr+No Drug, **P<0.001 vs Scr+TGF-β, ^#^*P*<0.05 vs Scr+No Drug; *n*=3 in each group.

**Fig. 4.**
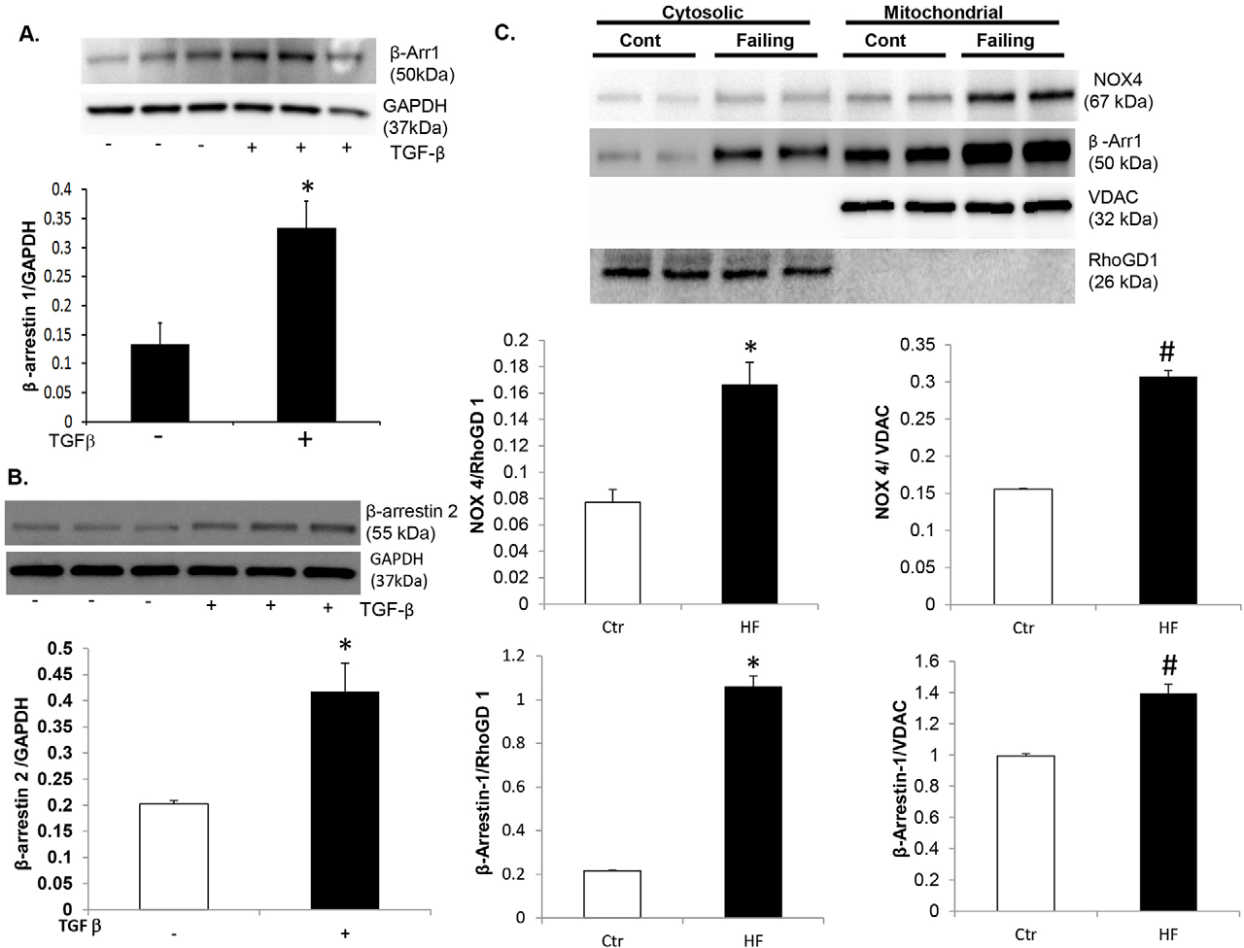
**β-arrestin is robustly expressed in fibroblast mitochondria.** (A) Representative immunoblot (upper panel) showing β-arrestin-1 expression under basal conditions and following TGF-β stimulation in control human cardiac fibroblasts (CFs). GAPDH was used as a loading control. Densitometric analysis (lower panel) demonstrates increased β-arrestin-1 expression in response to TGF-β in CFs. *P<0.004 vs No Drug. *n*=3 in each group. (B) Representative immunoblot (upper panel) showing β-arrestin-2 expression under basal conditions and following TGF-β stimulation in control human CFs. GAPDH was used as a loading control. Densitometric analysis (lower panel) demonstrates increased β-arrestin-2 expression in response to TGF-β in CFs. *P<0.01 vs No Drug. *n*=3 in each group. (C) Cytosolic and mitochondrial fractionations were isolated from control and failing human CFs. Representative immunoblot (upper panel) showing Nox4 and β-arrestin-1 expression in cytosolic and mitochondrial fractions of normal (Cont) and failing human CFs. This blot was stripped and re-probed for RhoGD1 and VDAC, which were used as cytosolic and mitochondrial loading controls, respectively. Densitometric analysis (middle panels) demonstrates increased Nox4 expression in both cytosolic (left) and mitochondrial (right) fractions in the failing (HF) CFs. *P=0.02 vs control in the cytosolic fraction and ^#^*P*=0.001 vs control in the mitochondrial fraction. Densitometric analysis (lower panels) demonstrates increased β-arrestin-1 expression in both cytosolic and mitochondrial fractions. *P=0.003 vs control in the cytosolic fraction and ^#^*P*=0.01 vs control in the mitochondrial fraction.

β-arrestins are scaffold proteins linking GPCR activation to multiple downstream signaling pathways. To identify a potential mechanism by which oxidative stress is regulated in failing CFs, we investigated the role of β-arrestins. Expression of both β-arrestin1 and 2 are increased over fourfold in failing CFs compared to controls as demonstrated by immunoblotting (Fig. 3A) and immunostaining (Fig. 3B). β-arrestin-1 and β-arrestin-2 expression was inhibited using an siRNA approach (Fig. 3C). Knockdown of either isoform resulted in significant inhibition of both basal and TGF-β-stimulated mitochondrial superoxide production (Fig. 3D). To further determine the mechanism by which β-arrestin inhibition decreased oxidative stress, we measured *Nox4* mRNA expression by quantitative PCR (qPCR). Knockdown of β-arrestin-1 (si-βarr1) led to a significant decrease in both basal and TGF-β-stimulated *Nox4* expression compared to the use of Scr (Fig. 3E). We also measured Nox4 expression at the protein level. Knockdown of either β-arrestin-1 (si-βarr1) or β-arrestin-2 (si-βarr2) led to a significant decrease in both basal and TGF-β-stimulated Nox4 expression compared to scrambled controls (Fig. 3F). Importantly, TGF-β stimulation in normal control CFs led to upregulation of β-arrestin-1 and -2 expression (Fig. 4A,B) and, because TGF-β signaling is significantly increased in the setting of HF and pathological remodeling, this provides another mechanism for increased β-arrestin activity. We also quantitated Nox4 expression in separate cellular compartments. The ratio of mitochondrial to plasma-membrane-bound Nox4 was approximately 2:1 in both control and failing CFs (Fig. 4C). Interestingly, β-arrestin expression was greater in the mitochondrial fraction relative to the cytosolic fraction under control and failing conditions and expression in both compartments was significantly upregulated in HF, compared with normal control CFs (Fig. 4C).

### β-arrestin overexpression increases mitochondrial superoxide production

**Fig. 5.**
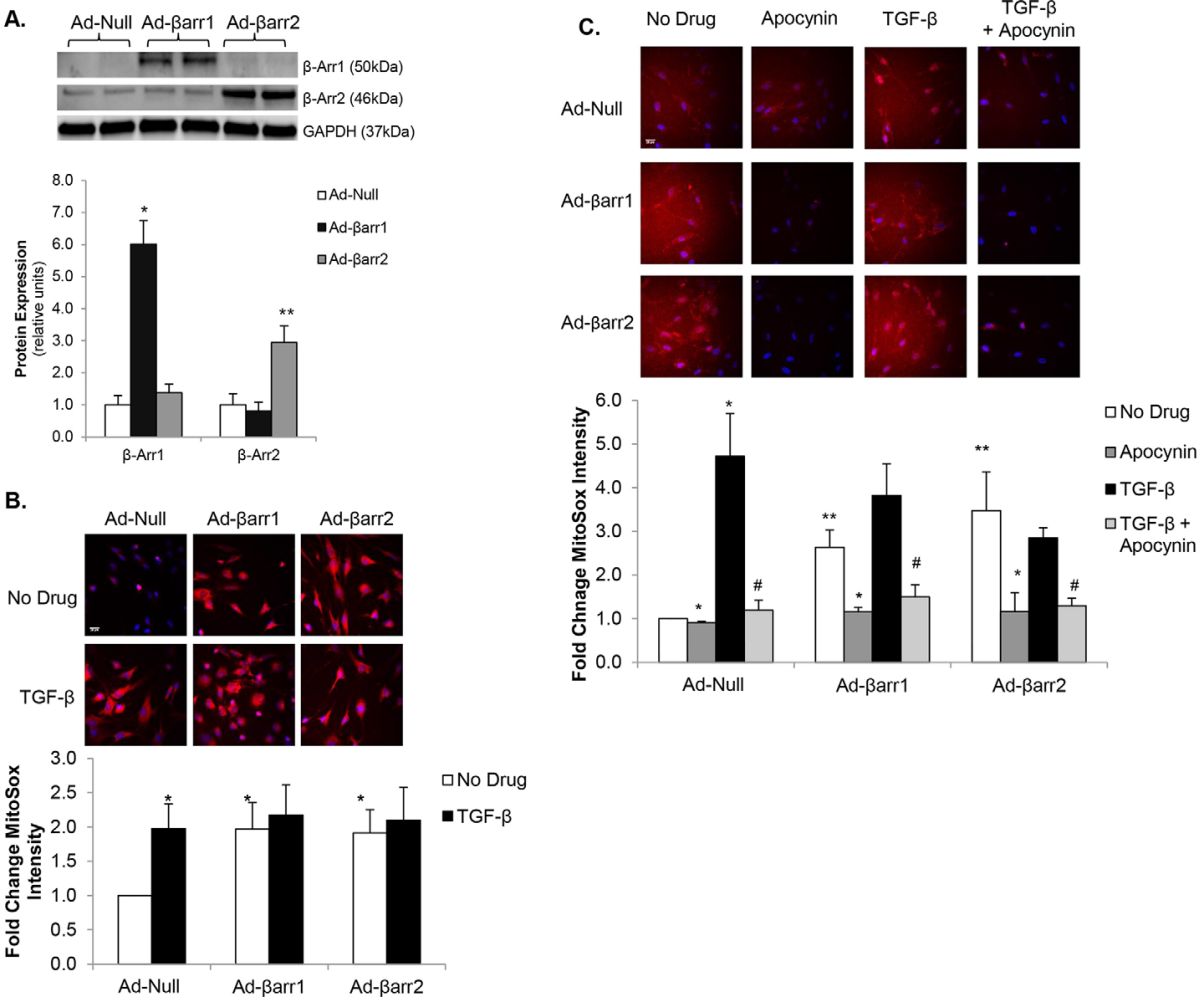
**β-arrestin overexpression increases mitochondrial oxidative stress.** (A) Representative immunoblot (upper panel) showing β-arrestin-1 (β-Arr1) and β-arrestin2 (β-Arr2) expression following transfection with adenoviruses overexpressing β-arrestin-1 (Ad-βarr1), β-arrestin-2 (Ad-βarr2), or control null virus (Ad-Null). GAPDH was used as a loading control. Densitometric analysis of β-arrestin-1 and β-arrestin-2 normalized to GAPDH expression is shown below. *P<0.001 vs Ad-Null and vs Ad-βarr2, **P<0.03 vs Ad-Null and vs Ad-βarr1; *n*=3-4 in all groups. (B) Confocal images (upper panel) of control cardiac fibroblasts (CFs) stained with MitoSOX (red) following transfection with Ad-βarr1, Ad-βarr2 or Ad-Null. Nuclei are stained blue with Hoechst 33342. Fluorescence quantitation is shown below. *P<0.05 vs Ad-Null+No Drug; *n*=3-5 in all groups. (C) Confocal images of transfected CFs stained with MitoSOX (red) showing mitochondrial oxidative stress under basal conditions, TGF-β stimulation, apocynin treatment, and pre-treatment with apocynin prior to stimulation with TGF-β (TGF-β+Apocynin). Nuclei are stained blue with Hoechst 33342. Fluorescence quantitation is shown below. *P<0.02 vs No Drug; **P<0.03 vs Ad-Null+No Drug; ^#^*P*<0.02 vs TGF-β; *n*=3 in each group. Scale bars: 20 μm.

We further investigated the role of β-arrestins in the regulation of CF oxidative stress utilizing adenoviral-mediated overexpression of β-arrestins in normal control CFs. First, we confirmed β-arrestin overexpression following adenoviral infection (Fig. 5A). Overexpression of β-arrestin-1 (Ad-βarr1) or β-arrestin-2 (Ad-βarr2) in control CFs led to a nearly twofold increase in mitochondrial superoxide levels as compared to a control null adenovirus (Ad-Null) (Fig. 5B). The increase in mitochondrial ROS generation following β-arrestin overexpression was similar to that following TGF-β stimulation and no additional increase in MitoSox staining was demonstrated following TGF-β stimulation with overexpression of either isoform. Stimulation with TGF-β following infection with Ad-βarr2 resulted in a 2.7-fold increase in mitochondrial superoxide levels that was not statistically different to either Ad-Null+TGF-β or Ad-βarr2 alone. To demonstrate more specifically that the increased oxidative stress in the setting of β-arrestin-1 or -2 overexpression is due to increased Nox4 activity, we treated control CFs with apocynin, a non-specific Nox inhibitor, following infection with Ad-βarr1, Ad-βarr2 or Ad-Null. Treatment with apocynin significantly decreased basal oxidative stress in both Ad-βarr1 and Ad-βarr2, reducing MitoSox staining to intensities similar to Ad-Null controls (Fig. 5C). Additionally, pre-treatment with apocynin significantly inhibited TGF-β-stimulated increases in mitochondrial superoxide production in all three groups. These data demonstrate that increased expression of β-arrestins to increases mitochondrial superoxide production in a Nox4-dependent manner.

### β-arrestin-stimulated oxidative stress is mediated by ERK

**Fig. 6.**
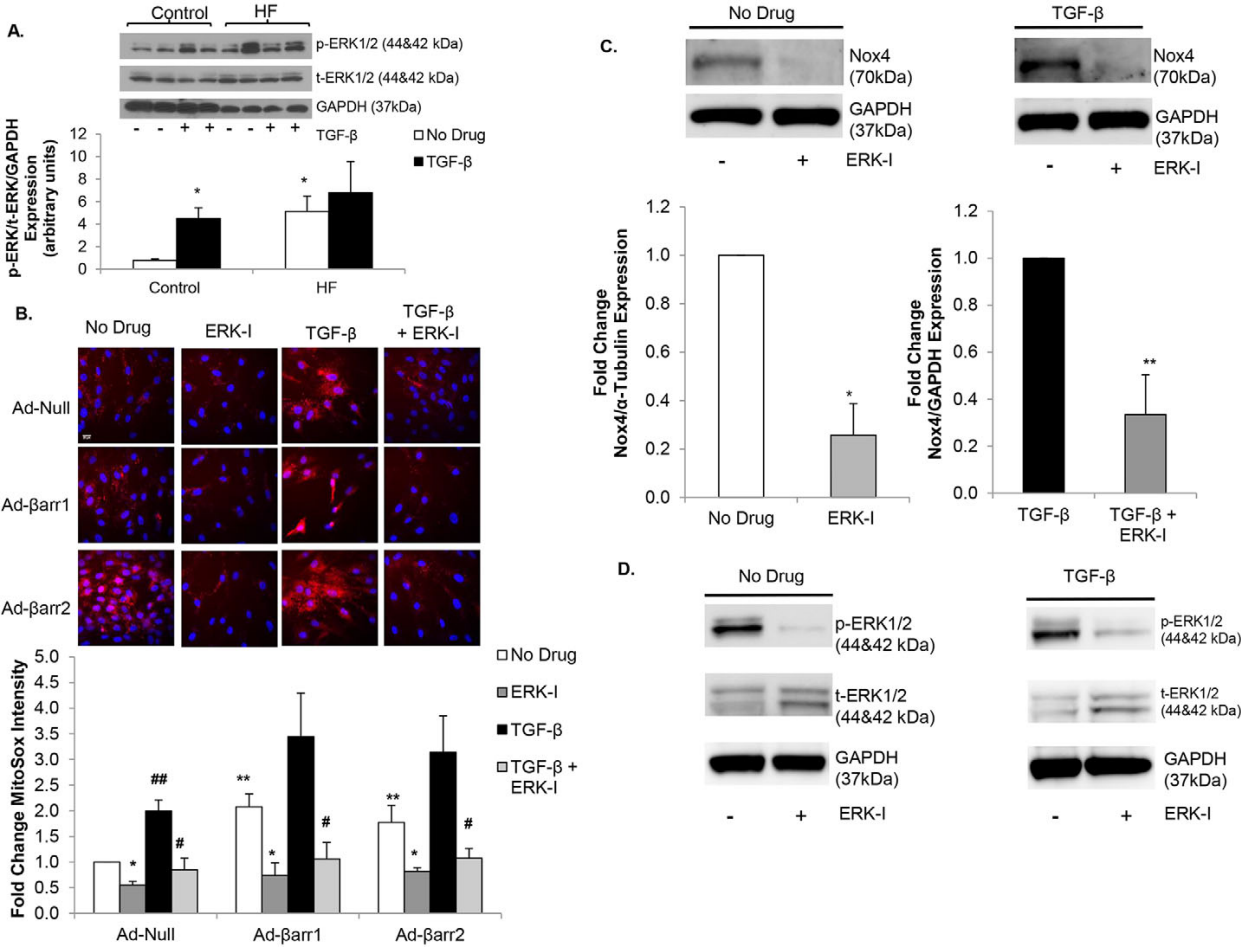
**β-arrestin-stimulated oxidative stress is mediated by ERK signaling.** (A) Representative immunoblot (upper panel) showing phosphorylated ERK1/2 (p-ERK1/2) and total ERK1/2 (t-ERK1/2) expression under basal conditions versus TGF-β stimulation in control and heart failure (HF) cardiac fibroblasts (CFs). GAPDH was used as a loading control. Densitometric analysis is shown below. *P<0.05 vs Control+No Drug; *n*=3-6 in all groups. (B) Confocal images (upper panel) of control CFs stained with MitoSOX (red) following transfection with adenoviruses overexpressing β-arrestin-1 (Ad-βarr1), β-arrestin-2 (Ad-βarr2) or control null virus (Ad-Null) under basal conditions, treatment with ERK inhibitor PD98059 (ERK-I), TGF-β-stimulation, and pre-treatment with PD98059 prior to stimulation with TGF-β (TGF-β+ERK-I). Nuclei are stained blue with Hoechst 33342. Fluorescence quantitation is shown below. *P<0.05 vs No Drug; **P<0.04 vs Ad-Null+No Drug, ^#^*P*<0.001 vs TGF-β, ^##^*P*=0.05 vs Ad-Null+No Drug; *n*=4 for all groups. Scale bar: 20 μm. (C) Representative immunoblots (upper panels) showing the effect of ERK inhibitor on basal (left panel) and TGF-β-stimulated (right panel) Nox4 expression in failing CFs. GAPDH was used as a loading control. Densitometric analysis is shown below. *P=0.01 vs No Drug, **P<0.03 vs TGF-β; *n*=3 in each group. (D) Representative immunoblots showing decreased basal (No Drug) (left panel) and TGF-β-stimulated (right panel) ERK1/2 phosphorylation following treatment with the ERK inhibitor (ERK-I) PD98059. GAPDH was used as a loading control.

**Fig. 7.**
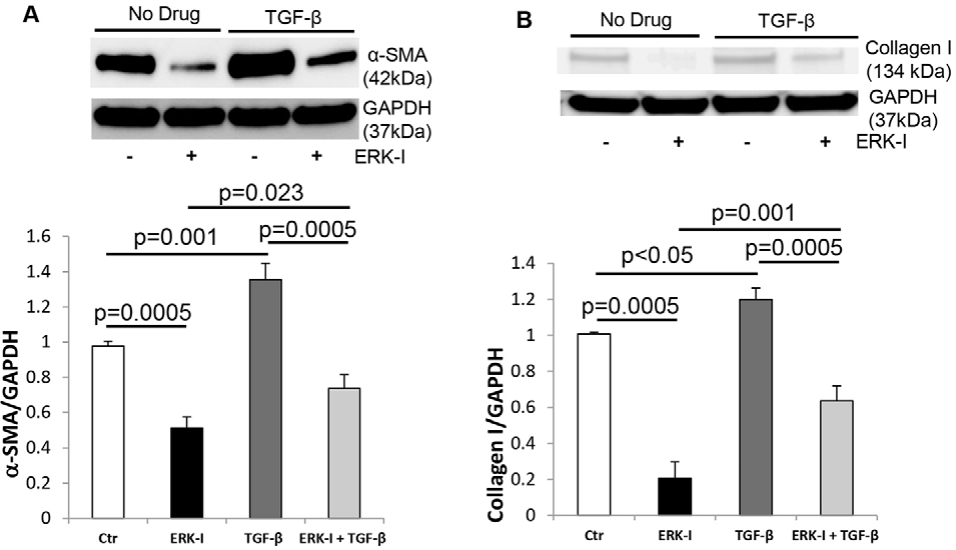
**NOX4/oxidative-stress-induced myofibroblast transformation is mediated by ERK signaling.** (A) Representative immunoblot (upper panel) showing α-SMA expression in control cardiac fibroblasts (CFs) under basal conditions (Ctr), TGF-β-stimulation, treatment with the ERK inhibitor PD98059 (ERK-I), and pre-treatment with PD98059 prior to stimulation with TGF-β (TGF-β+ERK-I). GAPDH was used as a loading control. Densitometric analysis is shown below. *n*=3 for all groups. (B) Representative immunoblot (upper panel) showing collagen I expression in control CFs under basal conditions, TGF-β-stimulation, treatment with the ERK inhibitor PD98059 (ERK-I), and pre-treatment with PD98059 prior to stimulation with TGF-β (TGF-β+ERK-I). GAPDH was used as a loading control. Densitometric analysis is shown below. *n*=3 for all groups.

TGF-β is known to increase CF myofibroblast differentiation and ECM production through activation of the MAPK/ERK pathway (Zhang, 2009). ERK phosphorylation was significantly increased in failing CFs versus controls (Fig. 6A). TGF-β stimulation increased ERK phosphorylation in control but not in failing CFs because the level of ERK phosphorylation was already maximal in the failing state (Fig. 6A). We investigated whether β-arrestin-stimulated increases in mitochondrial superoxide levels were ERK-mediated. Control CFs were infected with Ad-βarr1, Ad-βarr2 or Ad-Null. Mitochondrial oxidative stress was quantified under basal conditions, after treatment with the ERK inhibitor PD98059 (ERK-I), following TGF-β stimulation, or with ERK-I treatment prior to TGF-β stimulation (TGF-β+ERK-I). ERK inhibition significantly decreased basal mitochondrial superoxide production in the setting of β-arrestin-1 or -2 overexpression (Fig. 6B). Additionally, ERK inhibition blunted any TGF-β-stimulated increases in mitochondrial superoxide levels (Fig. 6B). To determine whether Nox4-mediated oxidative stress in failing CFs could be due to ERK activation, we measured Nox4 expression following treatment with the ERK-I. ERK inhibition diminished basal and TGF-β-stimulated Nox4 expression (Fig. 6C). Fig. 6D confirms that ERK1/2 phosphorylation was blunted by ERK-I treatment. To determine whether ERK phosphorylation is crucial for Nox4-mediated myofibroblast transformation, we measured α-SMA and collagen I expression under basal conditions, after treatment with the ERK-I, following TGF-β stimulation, or with ERK-I treatment prior to TGF-β stimulation (TGF-β+ERK-I) in control fibroblasts. ERK inhibition significantly decreased basal α-SMA and collagen I expression, and also decreased TGF-β-stimulated α-SMA and collagen I production (Fig. 7A,B). These studies demonstrate that TGF-β- and β-arrestin-stimulated increases in oxidative stress are ERK-mediated and provide evidence that increased mitochondrial superoxide production in CFs, in the setting of HF, might be regulated by increased ERK activity.

## DISCUSSION

Our data demonstrate significant upregulation of mitochondrial superoxide production and Nox4 expression in adult CFs isolated from failing human LV compared to non-failing controls. Inhibition of Nox4, either by treatment with apocynin or via siRNA-mediated knockdown, decreased transformation to activated myofibroblasts, as measured by α-SMA expression, and decreased collagen synthesis in both failing and control CFs. Our data also show that upregulation of Nox4 and increased mitochondrial oxidative stress contribute to CF-mediated cardiac fibrosis and remodeling. TGF-β treatment led to significant increases in Nox4 expression and mitochondrial superoxide generation. TGF-β-stimulated myofibroblast differentiation and collagen synthesis were also demonstrated to be Nox4-dependent. MitoSOX fluorescence is a well established and widely utilized method for quantification of mitochondrial superoxide generation. Our data provide clear evidence that changes in Nox4 expression significantly influence mitochondrial oxidant levels as measured by MitoSOX.

β-arrestin-1 and -2 are upregulated in CFs in the setting of HF. Knockdown of either β-arrestin isoform decreased both Nox4 expression and mitochondrial superoxide production. Furthermore, overexpression of β-arrestins led to a significant increase in mitochondrial superoxide levels through upregulation of Nox4 expression. The ERK inhibitor studies demonstrate that β-arrestin and TGF-β potentially drive increases in Nox4 expression and mitochondrial ROS through ERK activation. These data provide additional evidence for the role of β-arrestins in regulating CF biology and for the potential novel therapeutic role of inhibiting Nox4-mediated mitochondrial ROS production through β-arrestins in the prevention of maladaptive cardiac remodeling and the development of HF. This study provides the first evidence that β-arrestins can regulate oxidative stress in adult human CFs via Nox4 in an ERK-dependent manner.

ROS production is elevated and contributes to the development of cardiac remodeling following myocardial infarction (Sun, 2009). There is substantial evidence that ROS activate MAPKs and NF-κB, accompanied by cardiac myocyte apoptosis, inflammation and interstitial fibrosis (Al Ghouleh et al., 2011). NADPH oxidases (Nox) are the major sources of ROS production in the myocardium in conditions such as myocardial infarction, HF and aging, and are therefore important contributors to the oxidative stress, cytokine release and cardiac dysfunction that underlie adverse myocardial remodeling (Kuroda et al., 2010; Ago et al., 2010). Nox4 is a major source of ROS in the setting of myocardial injury and plays a crucial role in activation of CFs and cardiac remodeling (Murdoch et al., 2006). Nox4 has been shown to be localized to the mitochondria and was found to be necessary for the development of pressure overload left ventricular hypertrophy in Nox4 transgenic and knockout mouse studies (Ago et al., 2010). Recent studies have also shown that inhibition of Nox4 by the plant phenolic alkaloid leonurine or an siRNA approach in neonatal rat CFs attenuated angiotensin-II-induced activation of ERK1/2, production of intracellular ROS, and expression of α-SMA and collagen I (Liu et al., 2013). Our data confirm the key role of ERK signaling in the upregulation of Nox4 and mitochondrial superoxide production.

Despite previous work on the role of Nox4 in maladaptive cardiac remodeling, mechanisms for the regulation of Nox4 expression and activity have been mostly unknown. β-arrestins are key signaling molecules involved in GPCR signaling. β-arrestin-1 and -2 are the primary isoforms expressed in the heart and target chronically activated GPCRs for internalization and degradation following phosphorylation by GPCR kinases (GRKs) (Kelly et al., 2008). Chronic HF is characterized by severely impaired β-adrenergic receptor (β-AR) signaling, with a 50% decline in total β-AR density and uncoupled signaling in the remaining receptors owing to increased expression and activity of GRK2 (Bristow et al., 1982; Ungerer et al., 1993). Inhibition of GRK2 in cardiac myocytes *in vitro* and animal models of HF *in vivo* has been shown to restore β-AR signaling and cardiac function (Dorn, 2009; Rengo et al., 2009). We recently demonstrated that inhibition of GRK2 in adult human failing CFs decreased myofibroblast transformation and collagen synthesis (D'Souza et al., 2011). It seems that β-arrestins regulate CF biology through Nox4/ROS-dependent mechanisms that seem to be ERK-mediated. It is well described that β-arrestin signaling also leads to activation of ERK (Lefkowitz et al., 2006). TGF-β is a very potent stimulus for ERK activation and ERK signaling has been demonstrated to stimulate collagen synthesis (Leask and Abraham, 2004; Blanchette et al., 2001). Thus, it seems that the upregulation of β-arrestin expression in HF contributes to increased ERK activation and subsequent upregulation of Nox4 and ROS generation in CFs.

In summary, this is the first report demonstrating upregulation of Nox4 and mitochondrial superoxide production in failing adult human CFs. This represents an important mechanism for CF-mediated myocardial fibrosis and adverse remodeling, a precursor to HF. Upregulation of Nox4 seems to be mediated by β-arrestins via ERK-dependent signaling. We also demonstrated increases in CF oxidative stress in the setting of TGF-β stimulation that are consistent with previous studies in normal adult mouse and human CFs. Furthermore, we provide evidence that both TGF-β and β-arrestin signaling through ERK promotes an increase in Nox4 expression and mitochondrial ROS generation. Further investigation of specific mechanisms for the ERK-mediated increase in Nox4 expression is required. In conclusion, inhibition of β-arrestin signaling in a CF-specific manner could represent a novel therapeutic approach to prevent pathological cardiac fibrosis.

## MATERIALS AND METHODS

All cell culture reagents were purchased from Invitrogen Technologies (Eugene, OR) except fetal bovine serum (FBS), which was obtained from Atlanta Biologicals (Lawrenceville, GA). Unless stated otherwise, all additional chemicals were obtained from Sigma-Aldrich (St Louis, MO). The anti-α-SMA and anti-vimentin antibodies were obtained from Sigma-Aldrich, collagen types I and III and β-arrestin-1 and -2 from Abcam (Cambridge, MA), phospho-p44/42 MAPK and ERK1/2 from Cell Signaling Technology, Inc. (Danvers, MA), and GAPDH and α-tubulin from Santa Cruz Biotechnologies (Santa Cruz, CA).

### Isolation and culture of adult human cardiac fibroblasts (CFs)

All procedures for tissue procurement in this study were performed in compliance with institutional guidelines for human research and with an approved Institutional Review Board protocol at the University of Chicago Medical Center and the University of Wisconsin School of Medicine and Public Health. These studies conform to the principles of the Declaration of Helsinki. Left ventricular (LV) tissue was taken from patients with severe LV dysfunction undergoing LV Assist Device (LVAD) implantation. The indication for LVAD in all patients (*n*=10) was end-stage heart failure (HF), defined as New York Heart Association functional class IV, with deterioration of cardiac function despite maximal medical therapy. Failing CFs were isolated by a modified method of D'Souza et al. (2011). Non-failing adult human LV CFs (control) were purchased from Cell Applications Inc. (San Diego, CA). Five different control fibroblast cultures were obtained. To prevent spontaneous differentiation, all studies were carried out in low serum (2.5% FBS) medium using early-passage cells (≤4) plated at a density of ∼200 cells per mm^2^. HF CFs were used within 2 weeks of culturing to ensure preservation of the failing phenotype.

### Protein immunoblotting

Protein immunoblotting was performed following the method described by D'Souza et al. (2011). Band intensity was quantitated using NIH ImageJ software. GAPDH or α-tubulin was used as a loading control.

### siRNA transfection of CFs

Target-specific siRNA duplexes were designed using the sequence from the open reading frame of human β-arrestins and *Nox4* mRNA to knockdown mRNA and protein expression of β-arrestins or Nox4. Human β-arrestin-1 (sc-29741, 5′-AAAGCCUUCUGCGCGGAGAAU-3′), β-arrestin-2 (sc-29208, 5′-AAGGACCGCAAAGUGUUUGUG-3′) and Nox4 (sc-41586) were obtained from Santa Cruz Biotechnology. Scrambled oligo-ribonucleotide complex was also obtained (sc-37007), which was not homologous to any mammalian genes and utilized as control (Scr). Cells were transfected with Lipofectamine 2000 (Invitrogen, Carlsbad, CA), according to the manufacturer's instructions. Silencing was quantified by immunoblotting and immunofluorescence. Only experiments with verified silencing were used.

### Real-time PCR analysis

Total RNA was isolated from cultured cells using Trizol reagent (Invitrogen) according to the manufacturer's protocol. RNA (1 μg) was used for the first-strand cDNA synthesis (Applied Biosystems, Carlsbad, CA). Quantitative RT-PCR was performed using the 7500 Fast Real-Time PCR System (Applied Biosystems). Each cDNA template was amplified in triplicate using SYBR Green PCR Master Mix (Applied Biosystems) with *Nox4* primers. *GAPDH* was used to normalize the value. The PCR primer sequences used are as follows: for human *Nox4* 5′-TGTCCTGCTTTTCTGGAAAACC-3 (forward), 5′-GTAGTCAGAATTGGCTTGGTCG-3 (reverse) and *GAPDH* 5′-AGACCACAGTCCATGCCATC-3′ (forward) and 5′-TTGCCCACAGCCTTGGCAG-3′ (reverse).

### Adenoviral infection of cell cultures

Control CFs were infected at a multiplicity of infection (MOI) of 10 with either an adenovirus encoding β-arrestin-1 (Ad-βarr1) or β-arrestin-2 (Ad-βarr2), or empty (Ad-Null) adenoviruses. The CFs were incubated with the virus for 18-24 h before stimulation with serum (DMEM/2.5% FBS) or TGF-β (10 ng/ml).

### Drug treatment protocol

CFs were grown to desired confluence in 5 ml of supplemented DMEM and treated with either TGF-β to reach a final concentration of 10 ng/ml or no drug in DMEM with 2.5% FBS. Cells were collected following 60 min, 24 h or 72 h of treatment. For Nox and ERK inhibitor studies, cells were treated with 100 μM apocynin or PD98059, respectively (Calbiochem, Billerica, MA), prior to stimulation with TGF-β or no drug.

### Collagen synthesis: [^3^H]proline incorporation

CFs were treated with TGF-β, no drug, PD98059, apocynin, or a combination as indicated in the figure legends. [^3^H]proline incorporation was measured according to the method described by Swaney et al. (2005) and D'Souza et al. (2011).

### Immunostaining and confocal microscopy

CF cells were grown on 12-mm coverslips and immunostaining was carried out following methods described by D'Souza et al. (2011) using the primary antibodies (and dilutions) against the following: Nox4 (1:50), vimentin (1:400), β-arrestin-1 and -2 (1:200). Cells were mounted in Fluoroshield™ with DAPI mounting medium (Sigma-Aldrich) and visualized using an Olympus DSU Spinning Disk Confocal.

### MitoSOX staining

Cells were grown on 0.3% gelatin-treated glass-bottom dishes. After siRNA or adenoviral transfection and drug treatment, cells were washed with Hank's balanced salt solution (HBSS). Cells were loaded with 5 μM MitoSOX Red (Invitrogen) and 1:1000 dilution of Hoechst 33342 (Invitrogen) in HBSS for 10 min at 37°C protected from the light. The cells were washed gently three times with warm buffer and placed in warm buffer for imaging. The indicator was detected using a confocal microscope at an excitation/emission maxima of 510/580 nm. The total fluorescence intensity per field was quantitated and standardized by total cell number using NIH ImageJ software analysis. The results are represented as fold change in relative fluorescence units.

### Statistical analysis

All data are expressed as mean±s.e.m. Student's *t*-test was used to analyze differences between experimental groups. Single sample *t*-test was used to analyze experimental groups for data represented as fold change from control. One-way or two-way ANOVA followed by Tukey's HSD post-hoc test were used as appropriate for experiments with three or more groups. Values of *P*<0.05 were considered significant.

## References

[DMM019968C1] Ago T., Kuroda J., Pain J., Fu C., Li H. and Sadoshima J. (2010). Upregulation of Nox4 by hypertrophic stimuli promotes apoptosis and mitochondrial dysfunction in cardiac myocytes. *Circ. Res.* 106, 1253-1264. 10.1161/CIRCRESAHA.109.213116 20185797 PMC2855780

[DMM019968C2] Al Ghouleh I., Khoo N. K. H., Knaus U. G., Griendling K. K., Touyz R. M., Thannickal V. J., Barchowsky A., Nauseef W. M., Kelley E. E., Bauer P. M. et al. (2011). Oxidases and peroxidases in cardiovascular and lung disease: new concepts in reactive oxygen species signaling. *Free Radic. Biol. Med.* 51, 1271-1288. 10.1016/j.freeradbiomed.2011.06.011 21722728 PMC3205968

[DMM019968C3] Baudino T. A., Carver W., Giles W. and Borg T. K. (2006). Cardiac fibroblasts: friend or foe? *Am. J. Physiol. Heart Circ. Physiol.* 291, H1015-H1026. 10.1152/ajpheart.00023.2006 16617141

[DMM019968C4] Blanchette F., Rivard N., Rudd P., Grondin F., Attisano L. and Dubois C. M. (2001). Cross-talk between the p42/p44 MAP kinase and Smad pathways in transforming growth factor beta 1-induced furin gene transactivation. *J. Biol. Chem.* 276, 33986-33994. 10.1074/jbc.M100093200 11448947

[DMM019968C5] Bristow M. R., Ginsburg R., Minobe W., Cubicciotti R. S., Sageman W. S., Lurie K., Billingham M. E., Harrison D. C. and Stinson E. B. (1982). Decreased catecholamine sensitivity and beta-adrenergic-receptor density in failing human hearts. *N. Engl. J. Med.* 307, 205-211. 10.1056/NEJM198207223070401 6283349

[DMM019968C6] Brown R. D., Ambler S. K., Mitchell M. D. and Long C. S. (2005). The cardiac fibroblast: therapeutic target in myocardial remodeling and failure. *Annu. Rev. Pharmacol. Toxicol.* 45, 657-687. 10.1146/annurev.pharmtox.45.120403.095802 15822192

[DMM019968C7] Byrne J. A., Grieve D. J., Bendall J. K., Li J.-M., Gove C., Lambeth J. D., Cave A. C. and Shah A. M. (2003). Contrasting roles of NADPH oxidase isoforms in pressure-overload versus angiotensin II-induced cardiac hypertrophy. *Circ. Res.* 93, 802-805. 10.1161/01.RES.0000099504.30207.F5 14551238

[DMM019968C8] Cucoranu I., Clempus R., Dikalova A., Phelan P. J., Ariyan S., Dikalov S. and Sorescu D. (2005). NAD(P)H oxidase 4 mediates transforming growth factor-β1-induced differentiation of cardiac fibroblasts into myofibroblasts. *Circ. Res.* 97, 900-907. 10.1161/01.RES.0000187457.24338.3D 16179589

[DMM019968C9] Dorn G. W. II (2009). GRK mythology: G-protein receptor kinases in cardiovascular disease. *J. Mol. Med.* 87, 455-463. 10.1007/s00109-009-0450-7 19229505

[DMM019968C10] D'Souza K. M., Malhotra R., Philip J. L., Staron M. L., Theccanat T., Jeevanandam V. and Akhter S. A. (2011). G protein-coupled receptor kinase-2 is a novel regulator of collagen synthesis in adult human cardiac fibroblasts. *J. Biol. Chem.* 286, 15507-15516. 10.1074/jbc.M111.218263 21357420 PMC3083230

[DMM019968C11] Kelly E., Bailey C. P. and Henderson G. (2008). Agonist-selective mechanisms of GPCR desensitization. *Br. J. Pharmacol.* 153 Suppl. 1, S379-S388. 10.1038/sj.bjp.0707604 18059321 PMC2268061

[DMM019968C12] Kinugawa S., Tsutsui H., Hayashidani S., Ide T., Suematsu N., Satoh S., Utsumi H. and Takeshita A. (2000). Treatment with dimethylthiourea prevents left ventricular remodeling and failure after experimental myocardial infarction in mice: role of oxidative stress. *Circ. Res.* 87, 392-398. 10.1161/01.RES.87.5.392 10969037

[DMM019968C13] Kuroda J., Ago T., Matsushima S., Zhai P., Schneider M. D. and Sadoshima J. (2010). NADPH oxidase 4 (Nox4) is a major source of oxidative stress in the failing heart. *Proc. Natl. Acad. Sci. USA* 107, 15565-15570. 10.1073/pnas.1002178107 20713697 PMC2932625

[DMM019968C14] Leask A. and Abraham D. J. (2004). TGF-beta signaling and the fibrotic response. *FASEB J.* 18, 816-827. 10.1096/fj.03-1273rev 15117886

[DMM019968C15] Lefkowitz R. J., Rajagopal K. and Whalen E. J. (2006). New roles for beta-arrestins in cell signaling: not just for seven-transmembrane receptors. *Mol. Cell* 24, 643-652. 10.1016/j.molcel.2006.11.007 17157248

[DMM019968C16] Liu X.-H., Pan L.-L., Deng H.-Y., Xiong Q.-H., Wu D., Huang G.-Y., Gong Q.-H. and Zhu Y.-Z. (2013). Leonurine (SCM-198) attenuates myocardial fibrotic response via inhibition of NADPH oxidase 4. *Free Radic. Biol. Med.* 54, 93-104. 10.1016/j.freeradbiomed.2012.10.555 23127783

[DMM019968C17] Murdoch C. E., Zhang M., Cave A. C. and Shah A. M. (2006). NADPH oxidase-dependent redox signalling in cardiac hypertrophy, remodelling and failure. *Cardiovasc. Res.* 71, 208-215. 10.1016/j.cardiores.2006.03.016 16631149

[DMM019968C18] Octavia Y., Brunner-La Rocca H. P. and Moens A. L. (2012). NADPH oxidase-dependent oxidative stress in the failing heart: from pathogenic roles to therapeutic approach. *Free Radic. Biol. Med.* 52, 291-297. 10.1016/j.freeradbiomed.2011.10.482 22080085

[DMM019968C19] Petrov V. V., Fagard R. H. and Lijnen P. J. (2002). Stimulation of collagen production by transforming growth factor-beta1 during differentiation of cardiac fibroblasts to myofibroblasts. *Hypertension* 39, 258-263. 10.1161/hy0202.103268 11847194

[DMM019968C20] Porter K. E. and Turner N. A. (2009). Cardiac fibroblasts: at the heart of myocardial remodeling. *Pharmacol. Ther.* 123, 255-278. 10.1016/j.pharmthera.2009.05.002 19460403

[DMM019968C21] Rengo G., Lymperopoulos A., Zincarelli C., Donniacuo M., Soltys S., Rabinowitz J. E. and Koch W. J. (2009). Myocardial adeno-associated virus serotype 6-betaARKct gene therapy improves cardiac function and normalizes the neurohormonal axis in chronic heart failure. *Circulation* 119, 89-98. 10.1161/CIRCULATIONAHA.108.803999 19103992 PMC2647661

[DMM019968C22] Sia Y. T., Lapointe N., Parker T. G., Tsoporis J. N., Deschepper C. F., Calderone A., Pourdjabbar A., Jasmin J. F., Sarrazin J. F., Liu P. et al. (2002). Beneficial effects of long-term use of the antioxidant probucol in heart failure in the rat. *Circulation* 105, 2549-2555. 10.1161/01.CIR.0000016721.84535.00 12034664

[DMM019968C23] Sun Y. (2009). Myocardial repair/remodelling following infarction: roles of local factors. *Cardiovasc. Res.* 81, 482-490. 10.1093/cvr/cvn333 19050008 PMC2639132

[DMM019968C24] Swaney J. S., Roth D. M., Olson E. R., Naugle J. E., Meszaros J. G. and Insel P. A. (2005). Inhibition of cardiac myofibroblast formation and collagen synthesis by activation and overexpression of adenylyl cyclase. *Proc. Natl. Acad. Sci. USA* 102, 437-442. 10.1073/pnas.0408704102 15625103 PMC544320

[DMM019968C25] Ungerer M., Bohm M., Elce J. S., Erdmann E. and Lohse M. J. (1993). Altered expression of beta-adrenergic receptor kinase and beta 1-adrenergic receptors in the failing human heart. *Circulation* 87, 454-463. 10.1161/01.CIR.87.2.454 8381058

[DMM019968C26] Weber K. T. (2004). Fibrosis in hypertensive heart disease: focus on cardiac fibroblasts. *J. Hypertens.* 22, 47-50. 10.1097/00004872-200401000-00011 15106793

[DMM019968C27] Zhang Y. E. (2009). Non-Smad pathways in TGF-beta signaling. *Cell Res.* 19, 128-139. 10.1038/cr.2008.328 19114990 PMC2635127

